# Seafood Object Detection Method Based on Improved YOLOv5s

**DOI:** 10.3390/s25247546

**Published:** 2025-12-12

**Authors:** Nan Zhu, Zhaohua Liu, Zhongxun Wang, Zheng Xie

**Affiliations:** 1 School of Physics and Electronic, Yantai University, Yantai 264005, China; zhunan@ytu.edu.cn (N.Z.); liuzhao_2001@126.com (Z.L.); 17853556157@163.com (Z.X.); 2 Shandong Provincial Data Open Innovation Application Laboratory for Advanced Smart Grid Technologies, Yantai University, Yantai 264005, China

**Keywords:** underwater target detection, deep learning, YOLOv5s

## Abstract

To address the issues of false positives and missed detections commonly observed in traditional underwater seafood object detection algorithms, this paper proposes an improved detection method based on YOLOv5s. Specifically, we introduce a Spatial–Channel Synergistic Attention (SCSA) module after the Fast Spatial Pyramid Pooling layer in the backbone network. This module adopts a synergistic mechanism where the channel attention guides spatial localization, and the spatial attention feeds back to optimize channel weights, dynamically enhancing the unique features of aquatic targets (such as sea cucumber folds) while suppressing seawater background interference. In addition, we replace some C3 modules in YOLOv5s with our designed three-scale convolution dual-path variable-kernel module based on Pinwheel-shaped Convolution (C3k2-PSConv). This module strengthens the model’s ability to capture multi-dimensional features of aquatic targets, especially in the feature extraction of small-sized and occluded targets, reducing the false detection rate while ensuring the model’s lightweight property. The enhanced model is evaluated on the URPC dataset, which contains real-world underwater imagery of echinus, starfish, holothurian, and scallop. The experimental results show that compared with the baseline model YOLOv5s, while maintaining real-time inference speed, the proposed method in this paper increases the mean average precision (mAP) by 2.3% and reduces the number of parameters by approximately 2.4%, significantly improving the model’s operational efficiency.

## 1. Introduction

With the continuous improvement of living standards and the deepening exploration of natural resources, the global demand for seafood is steadily increasing. In response, China has elevated its strategy of becoming a maritime power to a national priority, emphasizing the importance of marine understanding and conservation. Traditional manual fishing operations not only pose significant risks and suffer from low efficiency but also contribute to marine pollution through discarded fishing gear and other harmful debris. Consequently, the adoption of advanced technologies to replace manual operations is of critical importance.

However, the underwater environment presents a series of unique challenges. Factors such as dynamic ocean currents, light refraction and scattering, and strong absorption by water severely degrade the image quality captured by underwater cameras—leading to color distortion, image blurring, and the loss of fine details. These issues critically impair the performance of existing underwater object detection models, hindering the accurate identification of seafood targets. Moreover, the inherent characteristics of seafood—such as high inter-class variability, diverse object sizes, frequent occlusions due to aggregation, strong visual similarity to backgrounds, and dynamic postures—further increase the likelihood of false detections and missed targets, making the detection task significantly more difficult.

To address these limitations, we propose an enhanced object detection framework based on YOLOv5s tailored for underwater seafood detection. Our method incorporates two key architectural innovations: (1) The first is a Spatial–Channel Synergistic Attention (SCSA) [1] module. The spatial attention captures the target position, and then the channel attention enhances the channel features of aquatic products. After dual screening, the interference of seawater background clutter is greatly reduced, and the model’s feature extraction capability is significantly improved. (2) The second is a three-scale convolution dual-path variable-kernel module based on Pinwheel-shaped Convolution (C3k2-PSConv) [2]. The Pinwheel-shaped multi-directional branches of PSConv extract multi-dimensional features of aquatic targets, such as horizontal, vertical, and diagonal features. Then, combined with the dual-branch parallel structure of the C3k2 module to further aggregate features, the model can also achieve complete target feature extraction for occluded targets and small targets through multi-directional feature complementation, which improves the model’s small target detection capability.

We validate our approach on the Underwater Robot Picking Contest (URPC) dataset, which includes four common marine species under challenging underwater conditions. Extensive experiments demonstrate that our improved YOLOv5s model significantly outperforms the baseline in terms of the mean average precision (mAP), precision, and recall while maintaining real-time inference speed.

## 2. Materials and Methods

Traditional object detection research has primarily focused on feature extraction and pattern classification techniques. Early detection methods typically relied on sliding window strategies to traverse entire images and generate candidate regions potentially containing objects. Classical descriptors such as Scale-Invariant Feature Transform (SIFT) [3] and Histogram of Oriented Gradients (HOG) [4] were employed to extract local features, followed by classifiers like Support Vector Machines (SVMs) [5] and Adaptive Boosting (AdaBoost) [6] to determine object presence and perform classification. Postprocessing steps, such as non-maximum suppression, were then applied to eliminate redundant bounding boxes and identify the optimal detection results. For example, Spampinato et al. (2008) successfully combined color and texture features for underwater species detection [7]. Oliver et al. (2010) investigated the impact of different underwater spread functions on feature detectors, highlighting the influence of imaging conditions on detection performance [8]. In 2018, Susanto et al. revealed the limitations of color-based features under varying illumination conditions, demonstrating the persistent challenges of underwater detection tasks [9]. These traditional approaches often suffer from high computational cost and prolonged processing times, making them less practical for real-time applications in dynamic underwater environments.

In recent years, deep learning-based object detection methods have become dominant and can be broadly categorized into two-stage and one-stage approaches. Two-stage detectors, such as R-CNN [10], Fast R-CNN [11], Faster R-CNN [12], and Mask R-CNN [13], first generate region proposals and then refine classification and localization. These models, particularly Mask R-CNN, which incorporates instance segmentation, show strong performance in complex underwater environments. In contrast, one-stage detectors like the YOLO series [14,15,16,17,18,19], SSD [20], and RetinaNet [21] perform classification and localization in a unified pipeline, offering improved inference speed suitable for real-time applications. YOLO models are especially renowned for their efficiency in underwater detection, while SSD leverages multi-scale feature maps for performance gains. RetinaNet introduces Focal Loss to address class imbalance in one-stage detection.

Several efforts have aimed to tailor these deep models for underwater scenarios. In 2019, Li et al. modified YOLO to support underwater mobile platforms [22]. In 2020, Liu et al. enhanced YOLOv3 with a GAN-based framework (UGAN-P) for marine organism detection [23], and Fan et al. proposed FERNet, a hybrid VGG-ResNet network, to boost feature representation [24]. In 2021, Peng et al. designed a sea cucumber detection approach using an improved feature pyramid network with shortcut connections [25]. In 2022, Knausgard et al. integrated SENet into YOLOv3’s backbone to improve fish detection accuracy, and Zhang et al. optimized YOLOv5 by refining K-means clustering for better anchor box generation [26]. In 2024, Cheng et al. proposed an underwater object detection algorithm (SPSM-YOLOv8) based on YOLOv8. By introducing SPDConv to replace ordinary standard convolution, the model’s feature extraction capability for aquatic products was significantly improved [27]. In 2025, Wang et al. proposed an enhanced lightweight model BSE-YOLO based on YOLOv10, and a Multi-Scale Attention Synergy Module was introduced to enhance the model’s perception of key features [28]. In 2025, Luo et al. proposed MAS-YOLOv11, and by introducing a Multi-Scale Dilated Attention Mechanism (MSDA), the model’s multi-scale detection capability for underwater objects was improved [29].

Despite these advancements, high-performance and accurate detection algorithms tailored to the unique challenges of underwater environments and the diverse characteristics of seafood remain scarce. Striking a balance between detection efficiency and precision in such scenarios continues to pose a significant technical challenge.

## 3. Methods

In object detection tasks, the YOLOv5s architecture is widely adopted due to its efficient and accurate performance. The model is composed of four main components: the input layer, backbone network, neck, and detection head. Each module plays a distinct and coordinated role in enabling high-performance object recognition. To address the challenges posed by underwater imagery—such as complex backgrounds, small object sizes, and irregular seafood shapes—we propose a series of structural improvements to YOLOv5s, significantly enhancing detection accuracy and robustness. This section details the design and functionality of the proposed improved YOLOv5s model.

The primary function of the input stage is to perform preprocessing operations, such as resizing, to ensure the smooth progression of the subsequent feature extraction process. The backbone network is responsible for extracting features from the input image. The Conv module performs convolution operations to extract spatial and channel information from the image. The SCSA and SPPF are both designed to enhance feature extraction capabilities. Specifically, the SCSA module achieves synergistic feature enhancement between spatial and channel dimensions, while the SPPF module utilizes multi-scale pooling techniques to achieve adaptive size output. The head output stage is responsible for predicting object categories, determining object locations, and evaluating confidence scores.

### 3.1. Network Architecture

**Figure 1:**
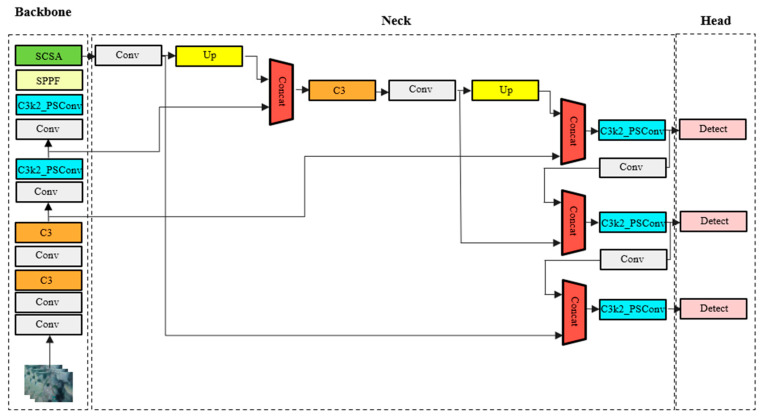
The improved YOLOv5s network structure.

To achieve high-precision and real-time object detection in practical scenarios, YOLOv5s adopts a streamlined yet robust architecture comprising four key components: the input module, backbone, neck, and detection head. The input module integrates adaptive image resizing and Mosaic data augmentation, which combines four randomly transformed images into a composite input. This not only improves robustness in scale variation but also enhances the model’s ability to detect small objects. Anchor boxes are automatically optimized for each dataset using k-means clustering followed by genetic evolution, eliminating the need for manual anchor tuning. The backbone features a combination of convolutional blocks (Conv), Cross-Stage Partial (C3) modules, and the Spatial Pyramid Pooling-Fast (SPPF) module, which efficiently captures hierarchical features across multiple receptive fields. In particular, SPPF replaces conventional multi-scale pooling with cascaded lightweight pooling operations, achieving richer spatial context at reduced computational cost. To further strengthen multi-scale feature fusion, the neck adopts a hybrid FPN-PAN structure that combines top-down semantic refinement with bottom-up localization enhancement, enabling the effective detection of small and densely packed objects. Both the backbone network and the neck network integrate the C3k2-PSConv module. First, the multi-directional branches of PSConv are used to enhance the features of aquatic targets and reduce interference from irrelevant features such as seawater and sediment. Then, the lightweight branches of C3k2 are adopted to further strengthen key features and suppress background clutter. The detection head performs bounding box regression using CIoU loss, which accounts for overlapping, center distance, and aspect ratio, and applies binary cross-entropy for classification and objectness prediction. Redundant detections are filtered using non-maximum suppression (NMS). These design choices collectively empower YOLOv5s to achieve an optimal trade-off between accuracy and efficiency, making it particularly well-suited for high-throughput tasks such as underwater seafood detection. The network structure diagram of the improved YOLOv5s model is shown in Figure 1.

### 3.2. Spatial–Channel Synergistic Attention (SCSA)

**Figure 2:**
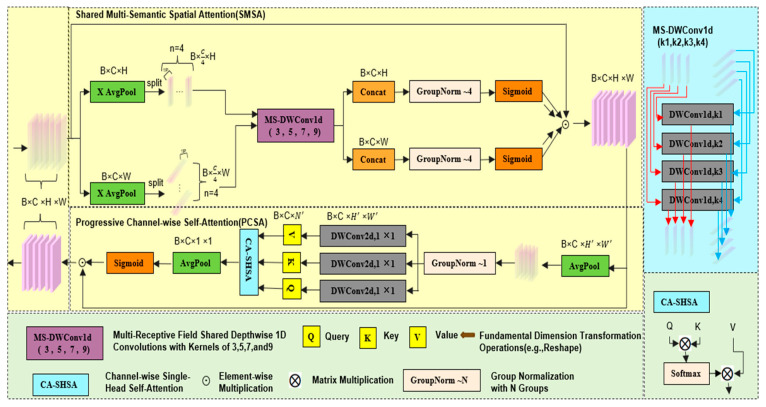
Structure diagram of SCSA.

To enhance the feature extraction capability of YOLOv5s for aquatic product targets, we employ an SCSA module, which is composed of two components: Shared Multi-Semantic Spatial Attention (SMSA) and Progressive Channel-wise Self-Attention (PCSA). The core operational principle of the SCSA module is to break the limitation of independent computation between spatial attention and channel attention and adopt a collaborative interaction approach to make the information of the two attention mechanisms mutually guide and enhance each other, thereby accurately capturing the key features of aquatic product targets. The structural diagram of SCSA is shown in Figure 2.

The SMSA module first performs dimension decomposition on the initial feature map $X\in R^{B\times C\times H\times W}$ (where *B* is the batch size, *C* is the number of channels, *H* is the height, and *W* is the width) along *H* and *W* using global average pooling, resulting in two one-dimensional sequences $X_{H}\in R^{B\times C\times W}$ and $X_{W}\in R^{B\times C\times H}$; subsequently, each of these two parts is divided into four identical sub-features ($X_{i}^{H}$ and $X_{i}^{W}$, i∈[1,4], and the number of channels for each sub-feature is C/4). The formulas for the sub-features are shown in (1) and (2):

(1) $$X_{i}^{H}=X_{H}[:,\left(i-1\right)\times C/4:i\times C/4,:]$$

(2) $$X_{i}^{W}=X_{W}[:,\left(i-1\right)\times C/4:i\times C/4,:]$$

After decomposing the feature map into sub-features, these 4 sub-features are, respectively, applied with depthwise separable one-dimensional convolutions (DWConv1d) with kernel sizes of *k_i_* = 3, 5, 7, 9 to capture information about different semantic spaces. Then, these sub-features with different semantics are concatenated (Concat). Subsequently, group normalization (GN) with 4 groups is used for processing. Finally, the Sigmoid activation function is employed and multiplied element-wise with the original feature map to obtain the spatial attention map. The corresponding formulas are shown in (3)–(7):

(3) $$Y_{i}^{H}={DWConv1d}_{k_{i}}^{C/4\to C/4}(X_{i}^{H})$$

(4) $$Y_{i}^{W}={DWConv1d}_{k_{i}}^{C/4\to C/4}(X_{i}^{W})$$

(5) $${Attn}_{H}=\sigma ({GN}_{H}^{4}(Concat(Y_{1}^{H},Y_{2}^{H},Y_{3}^{H},Y_{4}^{H})))$$

(6) $${Attn}_{W}=\sigma ({GN}_{W}^{4}(Concat(Y_{1}^{H},Y_{2}^{H},Y_{3}^{H},Y_{4}^{W})))$$

(7) $$SMSA\left(X\right)=X_{S}={Attn}_{H}{\;\times \;Attn}_{W}\;\times \;X$$

where σ(.) is the Sigmoid activation function; ${GN}_{H}^{4}$(.) and ${GN}_{W}^{4}$(.) are 4-group GN operations along the *H* and *W* dimensions, respectively; and *X_S_* is the final spatial attention map.

To preserve the multi-semantic spatial information of SMSA, the PCSA module adopts a progressive compression method based on average pooling, which can effectively utilize the spatial information provided by SMSA for deep learning. Finally, a serial structure is used to integrate the SMSA and PCSA modules, and the constructed SCSA module significantly improves the feature extraction capability of the model. The formulas of the PCSA module and the SCSA module are shown in (8)–(13):

(8) $$X_{p}={Pool}_{\left(7,7\right)}^{\left(H,W\right)\to \left(H^{\prime },W^{\prime }\right)}(X_{s})$$

(9) $$F_{proj}={DWConv1d}_{\left(1,1\right)}^{C\to C}$$

(10) $$Q=F_{proj}^{Q}\left(X_{p}\right),K=F_{proj}^{K}\left(X_{p}\right),V=F_{proj}^{V}\left(X_{p}\right)$$

(11) $$X_{attn}=Attn\left(Q,K,V\right)=Softmax(\frac{QK^{T}}{\sqrt{C}})V$$

(12) $$PCSA\left(X_{s}\right)=X_{c}=X_{s}\times \sigma ({Pool}_{\left(H^{\prime },W^{\prime }\right)}^{\left(H^{\prime },W^{\prime }\right)\to \left(1,1\right)}(X_{attn}))$$

(13) $$SCSA\left(X\right)=PCSA(SMSA(X))$$

where ${Pool}_{\left(k,k\right)}^{\left(H,W\right)\to \left(H^{\prime },W^{\prime }\right)}$ denotes a pooling operation with a kernel size of (*k*,*k*) (.), which can adjust the resolution of the feature map from (H,W) to ($H^{\prime },W^{\prime }$); $F_{proj}$(.) represents a linear projection operation, which is used to generate queries (query, Q), keys (key, K), and values (value, V).

### 3.3. Three-Scale Convolution Dual-Path Variable-Kernel Module Based on Pinwheel-Shaped Convolution (C3k2-PSConv)

**Figure 3:**
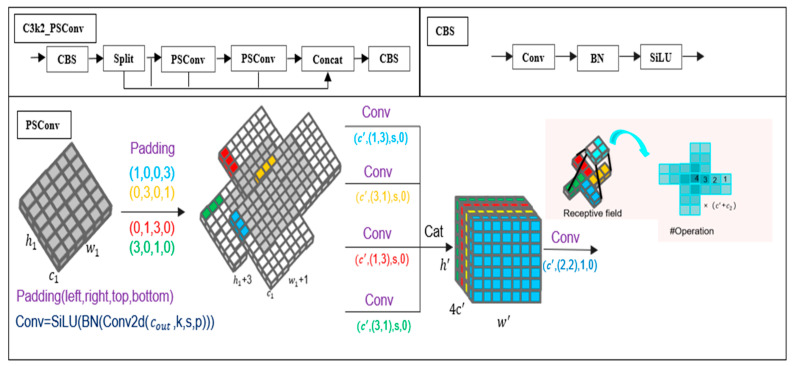
Structure diagram of C3k2_PSConv.

To enhance the model’s capability in detecting small aquatic product targets, we introduce C3k2-PSConv, as illustrated in Figure 3. C3k2-PSConv first goes through the CBS module, and then a splitting operation is performed to divide the feature map into two parts. Subsequently, it passes through two PSConv modules, and finally, the two parts of the feature maps are concatenated and output to the CBS module. C3k2-PSConv not only retains the feature reuse and efficiency advantages of C3k2 but also can accurately capture the features of small aquatic product targets via PSConv.

The PSConv module first employs asymmetric padding on the initial feature map X and uses horizontal (1 × 3) and vertical (3 × 1) convolution kernels to extract features in parallel. After that, 4 feature maps undergo grouped convolution and concatenation operations, and then a convolution kernel $W^{\left(2,2,c_{2}\right)}\;$ is utilized to enhance key feature channels and control the number of parameters. *h*, *w*, and *c* and *h′*, *w′*, and *c′* are the height, width, and number of channels of the initial and output feature maps, respectively. The formulas of PSConv are shown in (14)–(21):

(14) $$X_{1}^{\left(h^{\prime },w^{\prime },c^{\prime }\right)}=SiLU(BN(X_{P(1,0,0,3)}^{\left(h_{1},w_{1},c_{1}\right)}⊗W_{1}^{\left(1,3,c^{\prime }\right)}))$$

(15) $$X_{2}^{\left(h^{\prime },w^{\prime },c^{\prime }\right)}=SiLU(BN(X_{P(0,3,0,1)}^{\left(h_{1},w_{1},c_{1}\right)}⊗W_{2}^{\left(3,1,c^{\prime }\right)}))$$

(16) $$X_{3}^{\left(h^{\prime },w^{\prime },c^{\prime }\right)}=SiLU(BN(X_{P(0,1,3,0)}^{\left(h_{1},w_{1},c_{1}\right)}⊗W_{3}^{\left(1,3,c^{\prime }\right)}))$$

(17) $$X_{4}^{\left(h^{\prime },w^{\prime },c^{\prime }\right)}=SiLU(BN(X_{P(3,0,1,0)}^{\left(h_{1},w_{1},c_{1}\right)}⊗W_{4}^{\left(3,1,c^{\prime }\right)}))$$

where, in Formulas (14)–(17), *P*(1, 0, 0, 3) represents the number of padded pixels in the left, right, upper, and lower directions of the image. ⊗ denotes the convolution operation, *BN*(.) stands for batch normalization, *SiLU*(.) is a linear activation operation, and $W^{\left(1,3,c^{\prime }\right)}$ is a 1 × 3 convolution kernel with the number of output channels being *c*′.

(18) $$h^{\prime }=h_{1}/s+1,w^{\prime }=w_{1}/s+1,c^{\prime }=c_{2}/4$$

(19) $$X^{\prime (h^{\prime },w^{\prime },4c^{\prime })}=Cat(X_{1}^{\left(h^{\prime },w^{\prime },c^{\prime }\right)},\ldots X_{4}^{\left(h^{\prime },w^{\prime },c^{\prime }\right)})$$

(20) $$h_{2}=h^{\prime }-1=h_{1}/s,w_{2}=w^{\prime }-1=w_{1}/s$$

(21) $$PSConv\left(X\right)=Y^{\left(h_{2},w_{2},c_{2}\right)}=SiLU(BN(X^{\prime (h^{\prime },w^{\prime },4c^{\prime })}⊗W^{\left(2,2,c_{2}\right)}))$$

where *Cat*(.) represents the concatenation operation; *h*_2_, *w*_2_, and *c*_2_ are the height, width, and number of channels of the final output feature map; s is the convolution stride; and $Y^{\left(h_{2},w_{2},c_{2}\right)}$ is the final output.

## 4. Experimental Results and Analysis

### 4.1. Environment Configuration

The experimental environment plays a crucial role in determining the efficiency and accuracy of model training. The proper configuration ensures the optimal utilization of hardware resources, accelerates training speed, and guarantees the reproducibility and reliability of experimental outcomes, which is essential for supporting subsequent research and practical deployment. The detailed hardware and software configuration used in our experiments is summarized in Table 1.

Hyperparameter settings are equally critical to model performance and generalization. Proper tuning can improve learning efficiency, enhance detection accuracy, reduce overfitting, and enable the model to adapt effectively to complex underwater scenarios. The hyperparameters used in our experiments are listed in Table 2.

### 4.2. Dataset

The Underwater Robot Picking Contest (URPC2020) dataset is specifically designed for underwater robot grasping tasks, aiming to advance the fields of underwater machine vision and robotics. The dataset focuses on object recognition, localization, and grasping tasks in underwater environments and provides a rich source for developing and evaluating detection algorithms under real-world conditions.

This paper adopts the official URPC2020 dataset, which contains 8501 underwater images and covers four primary categories of marine objects: echinus, starfish, holothurian, and scallop. The dataset captures a wide range of underwater variations in illumination, turbidity, and scene dynamics. For training and evaluation, the dataset was split into a 7:2:1 ratio: training set—6320 images; validation set—1223 images; and test set—958 images.

**Figure 4:**
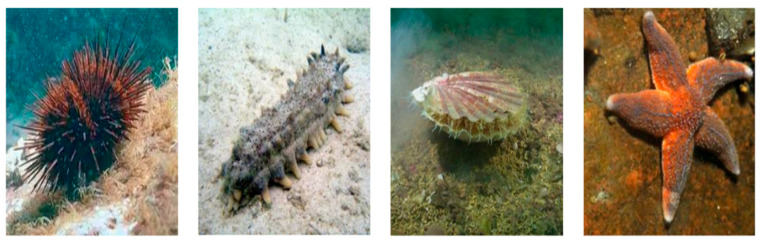
Sample images of four seafood categories.

We used the official URPC2020 dataset annotations and did not re-annotate. Figure 4 displays sample images of the four seafood categories. It can be seen that echinus and starfish have irregular shapes, and their underwater target features are relatively obvious and easy to recognize. However, holothurian and scallop are often in underwater sediment, and their underwater visual targets are not obvious, making recognition difficult.

### 4.3. Evaluation Metrics

To comprehensively evaluate the performance of our detection model, we adopt six commonly used metrics. In classification-based predictions, True Positive (TP) refers to the number of positive samples correctly identified as positive. False positive (FP) indicates the number of negative samples incorrectly predicted as positive, reflecting false alarms. True Negative (TN) represents correctly identified negative samples, while False Negative (FN) refers to positive samples that are mistakenly classified as negative, indicating missed detections. Precision (P) and recall (R) are defined as shown in Equations (22) and (23):

(22) $$P=\frac{TP}{\left(TP+FP\right)}$$

(23) $$R=\frac{TP}{TP+FN}$$

To further assess model accuracy, we construct a precision–recall (PR) curve using recall as the *x*-axis and precision as the *y*-axis. The average precision (AP) is defined as the area under the PR curve, as shown in Equation (24).

(24) $$\begin{array}{c} AP=\int_{0}^{1}PRdR \end{array}$$

The mean average precision (mAP) is the mean of AP scores over all object classes, where *n* is the total number of categories, as defined in Equation (25).

(25) $$\begin{array}{c} mAP=\frac{1}{n}\sum_{i=1}^{n}AP_{i} \end{array}$$

### 4.4. Analysis of Experimental Results

#### 4.4.1. Impact of Different Improvement Strategies on Detection Performance

The purpose of the ablation study is to assess the contribution of each component within the proposed model by systematically removing or modifying specific modules. This process enables a clearer understanding of how individual design choices affect overall detection performance. As shown in Table 3, we conduct ablation experiments on the improved YOLOv5s-based seafood detection model to evaluate the impact of key architectural enhancements.

In this ablation study based on the YOLOv5s model, we progressively introduce the SCSA and C3k2-PSConv modules to evaluate their individual contributions as well as their combined effect on detection performance. First, incorporating the SCSA module into the backbone network leads to an increase in mAP to 0.9%. This is mainly due to the collaborative mechanism of SCSA, which can focus on local key features. It first detects the unique features of aquatic product targets through channel attention and then actively amplifies the features of this region through spatial attention. Under the synergistic feature enhancement of “channel details + spatial positioning”, it accurately captures the key features of targets and improves the feature extraction ability of the model. When some C3 modules in the model are replaced with C3k2-PSConv, the mAP of the model is increased by 1.4%. This indicates that C3k2-PSConv can adopt the characteristics of “asymmetric padding + multi-direction convolution” to accurately extract the shape features of small organisms such as sea cucumbers and sea urchins and improve the small target detection ability of the model. When the two modules are jointly integrated into the model, compared with the original model, mAP is increased by 2.3%. The parameter count is reduced by approximately 2.4% compared to the original model, and both precision and recall are improved. This result proves that the SCSA and C3k2-PSConv modules are complementary, and the two achieve a synergistic effect of feature enhancement while ensuring lightweightness. In addition, this study uses YOLOv10n and YOLOv11n as baseline models and conducts ablation experiments by integrating the SCSA and C3k2-PSConv modules, respectively. As can be seen from the data in the table, after introducing the above two modules, although the number of parameters of YOLOv10n and YOLOv11n is slightly reduced, the precision (P), recall (R), and mean average precision (mAP) all decrease rather than increase. This result indicates that the latest lightweight network models did not achieve ideal performance improvements under the improvement strategy of this study and also verifies that such models are not suitable for the target recognition scenario of aquatic products.

#### 4.4.2. Qualitative Comparison

**Figure 5:**
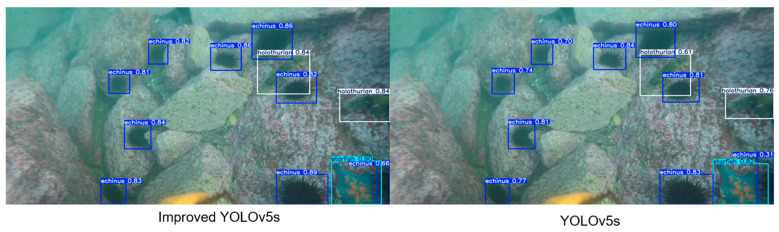
Test images of the improved model and the benchmark model in multi-target scenarios.

**Figure 6:**
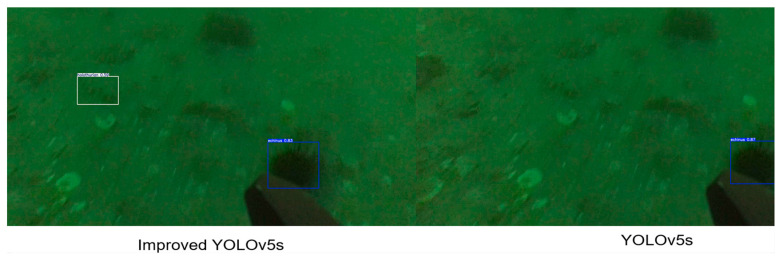
Test images of the improved model and the benchmark model in turbid water scenarios.

To evaluate the effectiveness of the proposed YOLOv5s algorithm improvement scheme, we trained the original version and the improved version of the model on the low-quality underwater dataset and tested their performance under the same conditions. As shown in Figure 5 and Figure 6, the detection results of the benchmark model (original YOLOv5s) and the improved YOLOv5s model in different underwater scenarios are compared. It can be seen from different images that detection difficulty increases significantly due to factors such as color distortion, the existence of a large number of small targets, and occlusion between aquatic products.

In Figure 5, the overall scene of the original image is relatively clear, but there are a large number of aquatic products, including nine sea urchins, two sea cucumbers, and one starfish. Both the benchmark model and the improved model can detect all aquatic product targets. However, by comparing the test images of the two models, it can be seen that the improved model has a higher confidence in target detection. Figure 6 presents the most challenging scene: The whole scene is dark green, and the image quality is blurry. The target size is small due to the long shooting distance, which further increases detection difficulty. There is only one sea urchin and one sea cucumber in the original image. The benchmark model fails to detect the sea cucumber mixed with sediment. The improved model, after introducing the C3k2-PSConv module, greatly improves its small target detection ability, and no missed detection occurs.

#### 4.4.3. Comparison with Different Models

To further demonstrate the superiority of the proposed model, we conduct comparative experiments against several mainstream object detection algorithms, including Faster R-CNN, SSD, YOLOv3, and YOLOv5s. The experimental results are presented in Table 4.

**Figure 7:**
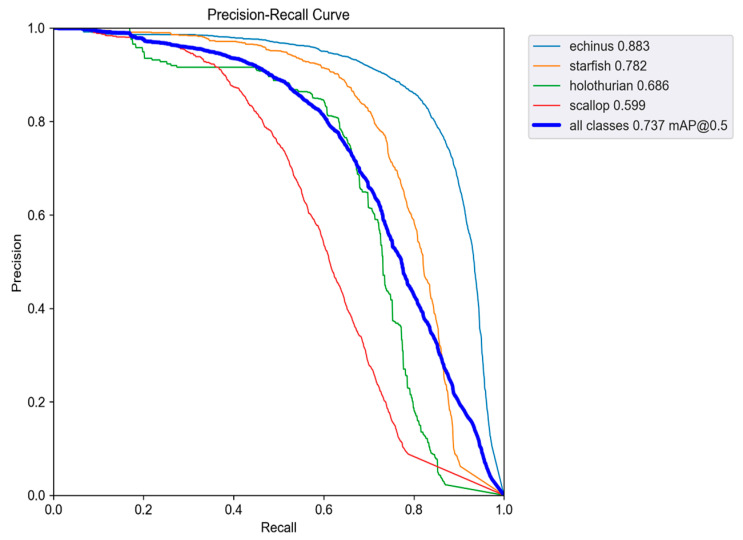
The Precision-Recall curve of the improved YOLOv5s.

As shown in Table 4, the proposed improved YOLOv5s model consistently outperforms existing mainstream detection algorithms across all four seafood categories. Compared to Faster R-CNN, SSD, YOLOv3, YOLOv8n, YOLOv10n, and YOLOv11n, our model achieves significantly higher average precision, particularly for challenging classes such as sea urchins and starfish. Even when compared to the original YOLOv5s, the improved model also achieves a significant improvement in both mAP and FPS; this proves the effectiveness and real-time performance of the integrated SCSA and C3k2-PSConv modules. These results confirm that the proposed enhancements substantially improve detection accuracy, robustness, and generalization in complex underwater environments, making the model well-suited for real-world seafood detection applications. The Precision-Recall curve of the improved YOLOv5s is shown in Figure 7.

#### 4.4.4. Category-Wise Detection Performance Analysis

We conduct a fine-grained performance analysis for each class. As shown in Table 5, we compare the original and improved YOLOv5s models on individual categories to assess class-specific improvements and detection consistency.

As can be seen from Table 5, the improved YOLOv5s model achieved stable improvements in precision (P) and recall (R) for all aquatic product categories. Notably, for categories with relatively vague underwater visual features such as holothurian and scallop, the improvement is significant, which indicates that the model’s detection ability for low-contrast targets was improved. For categories with relatively obvious underwater visual features and easy recognition such as echinus and starfish, the improvement effect is relatively moderate, but it also indicates that the improved model has more accurate recognition ability for clear targets. Overall, the improved model achieved a better balance between precision and recall in all categories, proving that it has excellent robustness and generalization ability in diverse underwater environments.

## 5. Conclusions

In this paper, we proposed an improved YOLOv5s-based object detection framework tailored for underwater seafood scenarios. To address the challenges of small object sizes, complex backgrounds, and visual ambiguity in underwater environments, we introduced two key architectural enhancements: the SCSA module and C3k2-PSConv. These two modules, while enhancing the model’s feature extraction capability and improving its small object detection ability, also reduce the computational overhead of the model.

Extensive experiments conducted on the URPC dataset demonstrate the effectiveness of the proposed approach. Ablation studies verify the individual and combined contributions of SCSA and C3k2-PSConv, with consistent improvements in the mean average precision (mAP). Qualitative comparisons further illustrate that the improved model shows a significant increase in detection accuracy under multi-object and low-light scenarios, with a notable reduction in missed detections. Moreover, comparative evaluations with mainstream detectors—including YOLOv8n, YOLOv10n, and YOLOv11n—highlight the superior detection performance of our method across all seafood categories. Category-wise analysis confirms that the model not only achieves higher detection accuracy but also maintains strong consistency across visually diverse target classes.

Overall, the proposed improvements to YOLOv5s lead to a more robust and precise detection system for underwater environments. The enhanced model demonstrates great potential for real-world applications in intelligent aquaculture, marine robotics, and automated seafood harvesting.

## Figures and Tables

**Table 1:** Environment setup for experiments.

Environment Component	Configuration Details
Operating System	Windows11
CPU	13th Gen Intel^®^ CoreTM i7-13650HX
GPU	NVIDIA GeForce RTX 4060
Framework	PyTorch2.0.1
CUDA Version	Cuda 12.6
Programming Language	Python 3.8

**Table 2:** Experimental parameter settings.

Parameter Name	Value
Batch Size	16
Number of Epochs	200
Input Image Size	640 × 640
Optimizer	Adam
Initial Learning Rate	0.01
Momentum	0.937
Weight Decay	0.0005
Warm-up Epochs	3

**Table 3:** Ablation experiment.

Baseline Model	SCSA	C3k2-PSConv	P(Precision)/%	R(Recall)/%	mAP(%)	Parameters (M)
YOLOv5s	×	×	78.8	63.3	71.4	7.02
	√	×	79.5	65.2	72.5	7.03
	×	√	79.1	65.5	72.8	6.83
	**√**	**√**	**80.1**	**66.3**	**73.7**	**6.85**
YOLOv10n	×	×	79.3	65.3	72.4	2.70
	√	×	78.7	64.0	70.2	2.59
	×	√	78.2	64.6	69.2	2.58
	**√**	**√**	**74.0**	**63.9**	**68.8**	**2.59**
YOLOv11n	×	×	78.7	64.5	71.9	2.59
	√	×	77.8	63.6	69.7	2.59
	×	√	76.7	63.3	69.3	2.45
	**√**	**√**	**74.1**	**62.8**	**68.5**	**2.45**

**Table 4:** Comparison with different models.

Model	AP(%)				mAP(%)	FPS (Frames per Second)
	Echinus	Starfish	Holothurian	Scallop		
Faster-RCNN	83.7	79.5	61.4	54.3	69.7	28.4
SSD	86.7	77.5	63.4	58.3	71.5	35.6
Yolov3	85.3	78.8	61.8	56.2	70.5	56.4
Yolov5s	87.1	77.1	65.4	55.2	71.2	221.7
Yolov8n	86.0	75.7	62.0	60.7	71.1	173.2
Yolov10n	85.4	76.0	67.0	61.5	72.4	169.5
Yolov11n	86.7	77.3	65.8	58.0	71.9	116.7
Yolov5s-Improve	88.3	78.2	68.6	59.9	73.7	225.3

**Table 5:** Analysis of detection results for different seafood categories.

Seafood Category	Algorithm	Precision (P)	Recall (R)
echinus	YOLOv5s	0.817	0.767
	YOLOv5s-Improve	0.841	0.82
starfish	YOLOv5s	0.825	0.748
	YOLOv5s-Improve	0.837	0.751
holothurian	YOLOv5s	0.751	0.572
	YOLOv5s-Improve	0.824	0.654
scallop	YOLOv5s	0.646	0.464
	YOLOv5s-Improve	0.797	0.475

## Data Availability

Data are available in a publicly accessible repository, link: https://aistudio.baidu.com/datasetdetail/228251 (accessed on 9 December 2025).
